# Rapid, Sensitive Detection of *Bartonella quintana* by Loop-Mediated Isothermal Amplification of the *groEL* Gene

**DOI:** 10.3390/ijms17121902

**Published:** 2016-12-01

**Authors:** Shoukui Hu, Lina Niu, Lijuan Luo, Xiuping Song, Jimin Sun, Qiyong Liu

**Affiliations:** 1 Clinical Laboratory of Peking University Shougang Hospital, Beijing100144, China; shoukuihua@163.com (S.H.); Luolyaduo@163.com (L.L.); 2 State Key Laboratory of Infectious Disease Prevention and Control, Collaborative Innovation Center for Diagnosis and Treatment of Infectious Diseases, National Institute for Communicable Disease Control and Prevention, Chinese Center for Disease Control and Prevention, Changping, Beijing 102206, China; songxiuping@icdc.cn (X.S.); jmsun@cdc.zj.cn (J.S.); 3 School of Tropical and Laboratory Medicine, Hainan Medical University, Haikou 571199, China; xiaoniuzhb@163.com; 4 School of Life Science, Shanxi University, Taiyuan 030006, China; 5 Zhejiang Provincial Center for Disease Control and Prevention, Hangzhou 310051, China

**Keywords:** *Bartonella*, trench fever, re-emerging pathogen, detection

## Abstract

Trench fever, caused by *Bartonella quintana*, is recognized as a re-emerging and neglected disease. Rapid and sensitive detection approaches are urgently required to monitor and help control *B. quintana* infections. Here, loop-mediated isothermal amplification (LAMP), which amplifies target DNA at a fixed temperature with high sensitivity, specificity and rapidity, was employed to detect *B. quintana*. Thirty-six strains, including 10 *B. quintana*, 13 other *Bartonella* spp., and 13 other common pathogens, were applied to verify and evaluate the LAMP assay. The specificity of the LAMP assay was 100%, and the limit of detection was 125 fg/reaction. The LAMP assay was compared with qPCR in the examination of 100 rhesus and 20 rhesus-feeder blood samples; the diagnostic accuracy was found to be 100% when LAMP was compared to qPCR, but the LAMP assay was significantly more sensitive (*p* < 0.05). Thus, LAMP methodology is a useful for diagnosis of trench fever in humans and primates, especially in low-resource settings, because of its rapid, sensitive detection that does not require sophisticated equipment.

## 1. Introduction

*Bartonella* are fastidious Gram-negative bacteria. They are transmitted by arthropods, for example lice and fleas are the vectors of *B. quintana* and *B. henselae*, respectively, to humans. They can cause several human diseases [1,2]. All *Bartonella* species are α-proteobacteria. Among them, *B. quintana* is the causative agent of trench fever and regarded as a re-emerging pathogen which infects humans and other primates [3]. Clinical manifestations include bacillary angiomatosis, chronic lymphadenopathy, endocarditis and trench fever [4,5,6]. Humans and primates are the major *B. quintana* reservoir and the human body louse has been considered the principal vector [7,8,9]. However, recently, this bacterium has been detected in specimens collected from cat fleas [10,11] and other arthropod families such as bed bugs [12,13], suggesting that a range of insects may act as vectors in the spread of trench fever. Human migration, habitat destruction, and changes in weather patterns or host dynamics increase the potential threat of sporadic and occasional epidemics of trench fever [14]. Hence, there is a need for rapid and specific methods to identify *B. quintana* and differentiate it from other *Bartonella* species to aid both diagnosis and treatment.

Diagnosis of trench fever remains challenging. Conventional methods for the isolation and identification of *B. quintana* require up to 4 weeks before they can be considered negative [15], which has obvious disadvantages in the clinical setting. Serological tests such as IFA (Indirect Immunofluoreseent, Assay, IFA) or immunoblotting are proven and renowned methods for diagnosis *Bartonella* infections. However, require paired samples from the acute and recovery phases and may not be useful for the diagnosis of acute disease [16]. PCR amplification of DNA is sensitive and specific, but requires sophisticated apparatus, which may not be available in in resource-poor settings, so this approach is impractical for diagnosis of *B. quintana* infection in many areas [17].

Loop-mediated isothermal amplification (LAMP) is a nucleic acid amplification method that can amplify up to 10^9^ copies of a DNA target in isothermal conditions (60–65 °C) in 1 h, and the results can be observed by a visual assessment of turbidity [18]. This assay has not only been applied to the detection of other bacterial species [19,20], but also to the detection of other *Bartonella* species other than *B. quintana* [21]. It is simpler than PCR-based methods and requires less equipment [22]. Here, we developed a LAMP assay targeting the molecular chaperone gene *groEL*, a member of the heat shock regulon, to detect *B. quintana*, and evaluated the diagnostic specificity and sensitivity of the assay.

## 2. Results

### 2.1. Confirmation and Detection of B. quintana Loop-Mediated Isothermal Amplification (LAMP) Reaction Products

**Figure 1:**
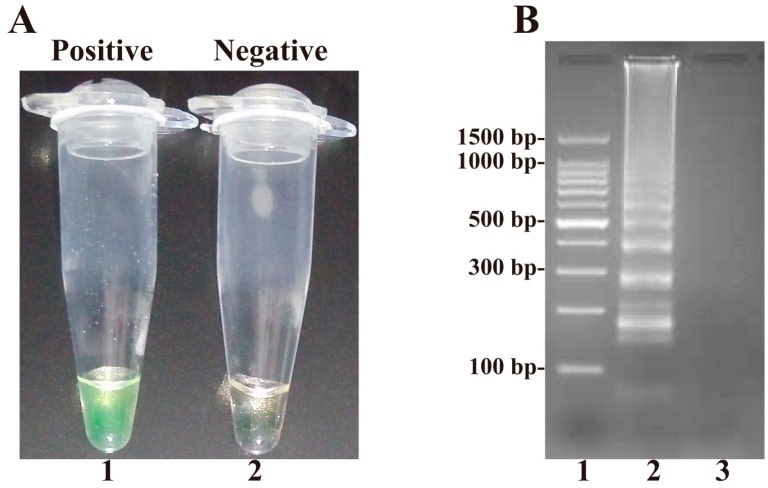
Result of the LAMP on detection of *B. quintana* (str. Toulouse): (**A**) color change of the LAMP; Tube 1 positive amplification; Tube 2 negative amplification; and (**B**) 2.5% agarose gel electrophoresis of LAMP product; Lane 1, DNA marker DL100-bp; Lane 2, LAMP product of *B. quintana*; Lane 3, negative control.

Amplification reactions were performed in the presence or absence of *B. quintana* genomic DNA to test the *B. quintana*-LAMP assay. Positive amplification was indicated by a color change from light gray to green, while the negative controls remained light gray (Figure 1A). After 2.5% agarose gel electrophoresis, positive reactions showed a ladder-liker pattern, but negative controls did not (Figure 1B).

### 2.2. The Optimal Temperature for the B. quintana LAMP Assay

**Figure 2:**
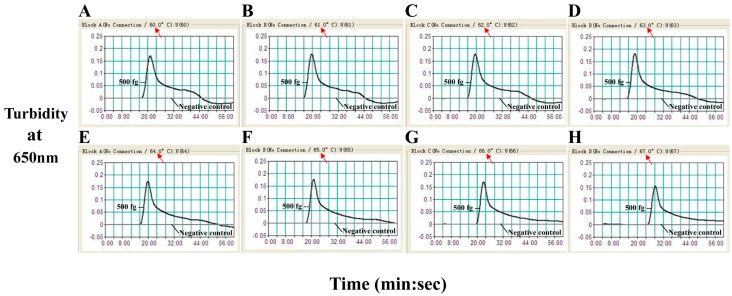
The optimal temperature for the LAMP assay. The LAMP amplifications reactions were analyzed by real-time measurement of turbidity and the corresponding curves of concentrations of DNA were marked in the Figure. The threshold value was 0.1 and the turbidity of >0.1 was considered to be positive. Eight kinetic graphs (**A**–**H**) were obtained at different temperature (60–67 °C) with *B. quintana* DNA at the level of 500 fg per reaction.

**Figure 3:**
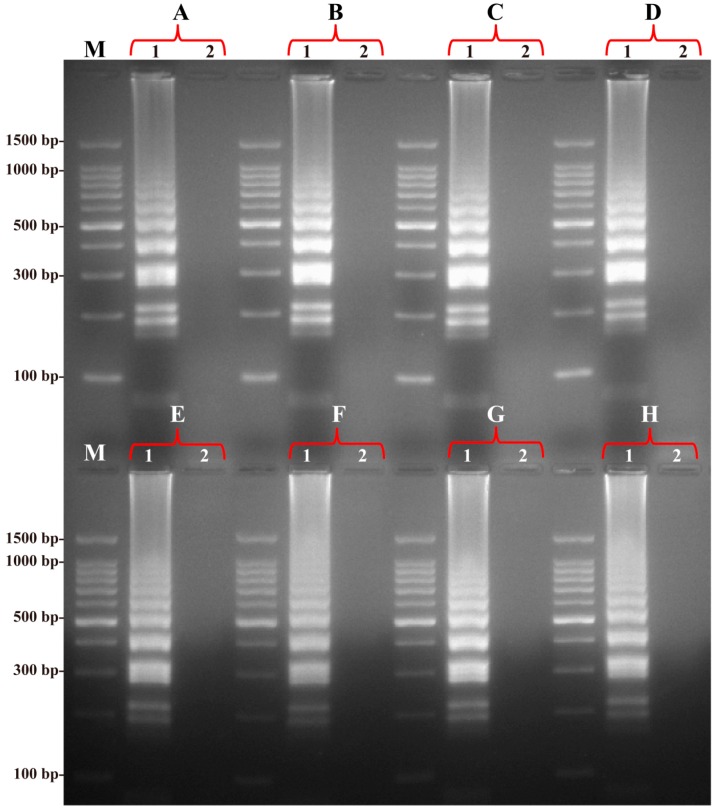
Products of the LAMP monitored using 2.5% agarose gel electrophoresis. The products (**A**–**H**) of the LAMP from different reaction temperature (60–67 °C) were monitored by 2.5% agarose gel electrophoresis after staining with ethidium bromide. Lane M, DL 100-bp DNA marker; Lane 1, positive LAMP products; Lane 2, negative control (no DNA).

The optimal temperature for the *B. quintana* LAMP reaction was determined using the reference strain *B. quintana* Toulouse as a positive control with 0.5 pg genomic DNA per reaction. The LAMP reactions were carried out at 60–67 °C and monitored by real-time turbidity measurement. Figure 2 shows typical kinetics, and Figure 3 shows agarose gel electrophoresis of the reaction products. A temperature of 63 °C was chosen as optimal for the LAMP reaction and used for the remainder of this study.

### 2.3. Specificity of the B. quintana LAMP Assay

**Figure 4:**
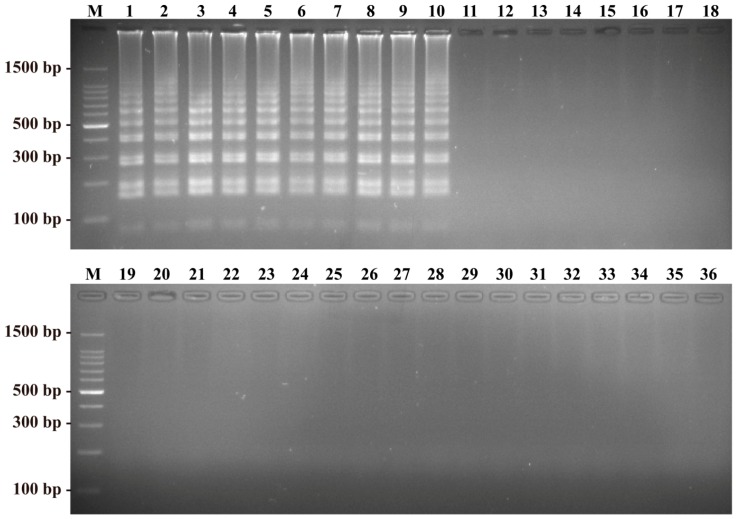
Specificity of the LAMP detection for different strains. Lane M, DL 50-bp DNA marker; Lane 1–10, different *B. quintana* strains; Lane 11–23, other *Bartonella* reference strains of *B. henselae*, *B. elizabethae*, *B. alsatica*, *B. koehlerae*, *B. vinsonii* subsp. *berkhoffii*, *B. vinsonii* subsp. *vinsonii*, *B. vinsonii* subsp. *arupensis*, *B. tribocorum*, *B. grahamii*, *B. clarridgeiae*, *B. bacilliformis*, *B. doshiae*, and *B. mayotimonensis*; Lane 24–36, *Yersinia enterocolitica*, *Pseudomonas aeruginosa*, *Enterococcus faecalis*, *Aeromonas hydrophila*, *Enterobacter sakazakii*, *Campylobacter jejuni*, *Bacillus cereus*, *Salmonella typhimurium*, *Vibrio cholera*, *Escherichia coli*, *Listeria Monocytogenes*, *Shigella sonnei*, and *Staphylococcus aureus*.

The assay specificity was determined with DNA templates from 23 members of the genus *Bartonella* (*B. quintana* 10 strains, other *Bartonella* spp. 13 strains) and 13 other common pathogenic bacteria. Reactions containing *B. quintana* genomic DNA produced a color change within 1 h, and a specific ladder of multiple bands observed after gel electrophoresis. No color change or bands on agarose gels were observed for the 23 non-*B. quintana* templates (Figure 4). Thus, the LAMP assay was highly specific for screening *B. quintana*.

### 2.4. Sensitivity of the B. quintana LAMP Assay

**Figure 5:**
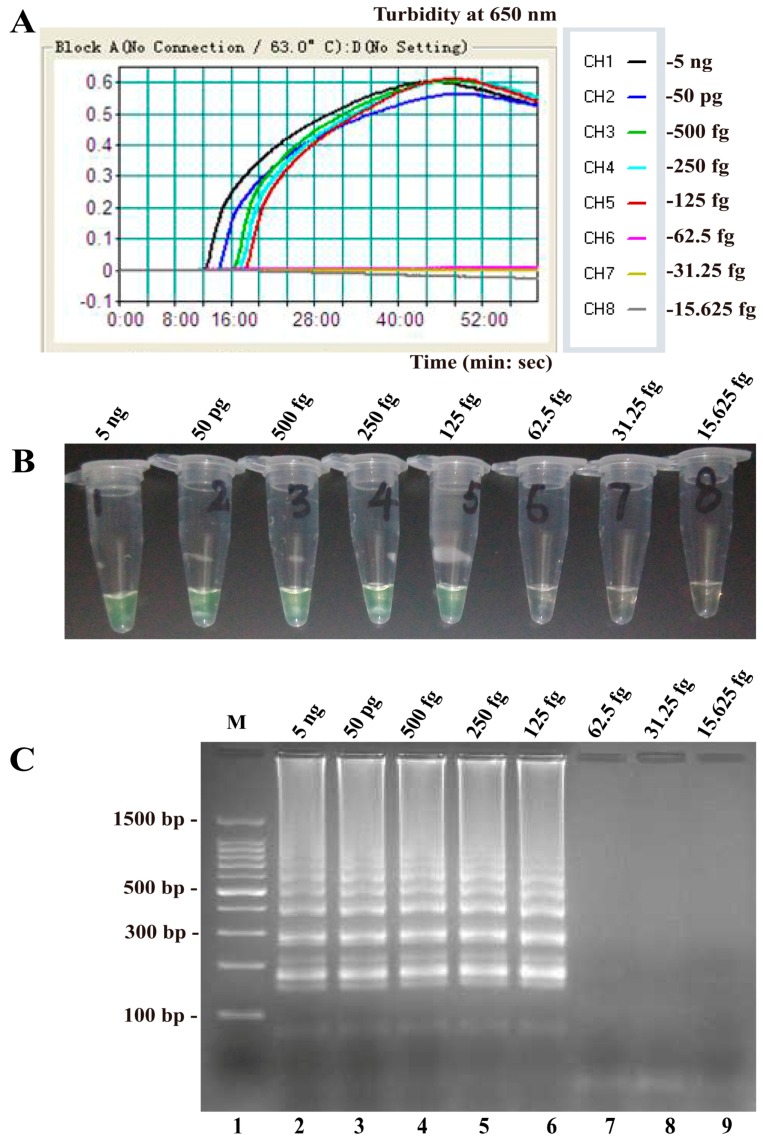
Sensitivity of the LAMP assay using serially diluted genomic DNA of *B. quintana* as template: (**A**) Sensitivity of the LAMP for *B. quintana* detection was analyzed by real-time measurement of turbidity; (**B**) The LoD for the LAMP assay was 125 fg genomic DNA per reaction; and (**C**) sensitivity of the LAMP for *B. quintana* detection wase seen using gel electrophoresis. Lane M, DL 50-bp DNA marker. The positive results were observed as a ladder-like pattern on 2.5% agarose gel electrophoresis analysis.

The LAMP assay was nearly fourfold more sensitive than qPCR for the detection of *B. quintana groEL* (Table 1), with detection limits of 125 and 500 fg DNA per reaction, respectively (Figure 5). *B. quintana* LAMP amplifications were monitored by real-time turbidity (Figure 5A) and agarose gel electrophoresis (Figure 5B,C). The LAMP reaction required incubation periods of 12, 14, 16, 17 and 18 min at genomic DNA levels of 5 ng, 50 pg, 500 fg, 250 fg and 125 fg per reaction, respectively (Figure 5A).

### 2.5. Evaluation of Practical Application of the B. quintana LAMP Assay

When testing 100 rhesus blood samples, we detected 22 positive results for *B. quintana* by the LAMP assay and eight by qPCR. The sensitivity of the LAMP assay was significantly higher than that of the qPCR (*p* < 0.05) (Table 2). Similarly, we tested 20 rhesus-feeder blood samples using these assays; the total positive rates were 20.0% for the LAMP assay (4/20) and 5.0% for the real-time PCR (1/20; *p* < 0.05) (Table 2).

## 3. Discussion

A novel LAMP assay to detect *B. quintana* by targeting the *groEL* gene was developed in this study. The limit of detection of the LAMP assay was 125 fg DNA/reaction, which was fourfold more sensitive than qPCR. No non-specific amplification or cross-reaction was observed when testing a panel of closely related bacteria (other *Bartonella* species) and common pathogens. In practical tests of rhesus blood samples and rhesus-feeder blood samples, the sensitivity of the LAMP assay was significantly higher (*p* < 0.05) than that of qPCR.

Trench fever caused by *B. quintana* is considered an important re-emerging infectious disease [23]. Once infected, patients suffer headache, recurrent fever, and pretibial pain. Due to persistent bacteremia [24], several major epidemics of trench fever occurred among soldiers in Europe during World Wars I and II. More recently, the disease has occurred occasionally in urban areas in Europe and the USA, mainly among the homeless, drug-addicts, and HIV-positive patients [4]. Thus, sensitive and accurate detection methods are required to monitor and study *B. quintana*.

Bacterial culture is the preferred method for identification of *Bartonella* infections, but is slow, labor-intensive and often poorly effective. PCR-based methods are a sensitive and selective approach to the detection of *Bartonella* and have been used to target genes including *gltA*, *rpoB*, the 16S–23S rRNA ITS, and *ftsZ* [25,26]. However, these techniques require a high-precision thermocycler, limiting their use in areas with basic clinical facilities, for example in rural endemic and impoverished areas. As an alternative, the LAMP method is rapid and simple to perform, requiring only a water bath or heating block for amplification. The test reaction can be performed within 18 min in isothermal conditions (in the present case 63 °C), enabling rapid molecular diagnosis [22,27]. Recent studies have shown that LAMP has high sensitivity and specificity compared to conventional, nested or qPCR for the detection of several intracellular bacteria, including *Coxiella burnetii* and *Orientiatsu tsugamushi* [28,29] and in distinguishing intraerythrocytic protozoan parasites, such as *Plasmodium* spp. and *Babesia* spp. [30,31]. The present study is the first report of a LAMP assay to identify *B. quintana*, and can be used in the field, clinic and veterinary laboratories in surveillance and diagnosis of trench fever.

## 4. Materials and Methods

### 4.1. Ethics

The protocol of this study was reviewed and approved by the Ethics Committee of the China Institute for Communicable Disease Control and Prevention, based on the medical research regulations of the Ministry of Health (Approval No. ICDC-2016003). All experimental procedures conformed to institutional guidelines for the care and use of laboratory animals as described by the China CDC, and to the National Institutes of Health Guide for Care and Use of Laboratory Animals (Publication No 85-23, revised 1985). Animal blood sampling was conducted with the consent of the animals’ owners.

### 4.2. Bacterial Strains and Culture Conditions

Thirty-six bacterial strains were used in this study, including 10 *B. quintana*, 13 other *Bartonella* species, and 13 non-*Bartonella* species (Table 3). All *Bartonella* strains were cultured on Tryptic Soy Agar (TSA) containing 10% sheep red blood cells using standard methods [32]. Other strains were cultured on Columbia agar containing 5% sheep blood at 37 °C for 24–48 h in an atmosphere containing 5% CO_2_. *B. quintana* str. Toulouse was used as the positive control to determine the optimal conditions for the LAMP assay and to establish baseline sensitivity. 

### 4.3. Genomic DNA Extraction

Bacterial genomic DNA was extracted from pure cultures using QIAamp DNA minikits (Qiagen, Hilden, Germany). The non-anticoagulated blood of rhesus macaque (2 mL) was collected, and the serum was separated by centrifugation at 3000× *g* for 10 min. Blood clots were used to extract DNA using the QIAamp DNA Mini Kit. All of the DNA samples from rhesus blood were tested using the LAMP assay, using 1 µL of the extracted DNA as the template. To verify the LAMP assay, a qPCR assay based on the *groEL* gene was used. The two methods were compared using the χ^2^ test and Fisher’s exact test. *p* < 0.05 was considered significant.

### 4.4. Design of LAMP Primers

**Figure 6:**
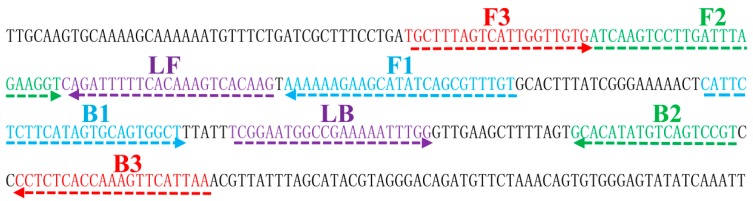
Location and sequences of *B. quintana groEL* (str. Toulouse) gene used to design the six primers. The sequences of the primer sites are underlined. Right and left arrows indicate sense and complementary sequences that are used.

Using the *groEL* sequence from *B. quintana* str. Toulouse (GenBank accession number: BX897700.1) and Primer Explorer v.4 software (http://primerexplorer.jp/), >100 LAMP primer sets were designed. A set of six specific primers was selected for LAMP to target eight distinct regions in *groEL*. Table 4 and Figure 6 show details of primer design, sequences, locations and target sequences.

### 4.5. LAMP Reaction

LAMP reactions were performed with the Loopamp Kit (Eiken Chemical Co., Ltd., Tokyo, Japan) in a 25-µL reaction containing 1.6 mM of each of the FIP (Forward Inner Primer) and BIP (Backward Inner Primer) primers, 0.8 mM of the LF (Loop Forward) and LB (Loop Backward) primers, 0.2 mM of the F3 and B3 primers, 12.5 µL 2× reaction mix, 1 µL of *Bst* DNA polymerase (8 U), 1 µL Loopamp Fluorescent Detection Reagent (FD), and 1 µL DNA template. The mixtures were incubated in a Loopamp Real-time Turbidimeter LA-320C (Eiken Chemical Co., Ltd.) at 63 °C for 60 min and then at 80 °C for 5 min to stop the reaction. Mixtures lacking DNA template were used as negative controls. After amplification, the positive LAMP products could be observed directly by the color change of the FD. Moreover, LAMP products were examined by 2.5% agarose gel electrophoresis. To determine the optimum reaction temperature, the LAMP amplification was carried out for 60 min at constant temperatures from 60 to 67 °C in 1 °C intervals, with final incubation for 5 min at 85 °C to terminate the reaction.

### 4.6. Specificity and Sensitivity of the B. quintana LAMP Assay

To assess specificity, LAMP reactions were carried out as described above using DNA templates from 23 *Bartonella* species and 13 non-*Bartonella* species (Table 3). Each sample was examined twice, independently. To determine the analytical sensitivity, genomic DNA from *B. quintana* str. Toulouse was serially diluted (to 5 ng, 50 pg, 500 fg, 250 fg, 125 fg, 62.5 fg, 31.25 fg, and 15.625 fg, respectively). The limits of detection of the LAMP and qPCR assays were ascertained from the lowest amount of template DNA that could be detected. Table 4 lists the primers and probe for the qPCR assay. qPCR amplification was performed in a 20-µL reaction containing 0.3 mM each primer, 0.2 mM probe, 10 µL PromegaGo*Taq*^®^ qPCR Master Mix, and 1 µL of DNA template. The PCR in an ABI PRISM system (Applied Biosystems, Carlsbad, CA, USA) involved predenaturation at 95 °C for 2 min, then 40 cycles of 95 °C for 3 s and extension at 60 °C for 30 s. Samples were tested in duplicate.

### 4.7. Practical Application of the B. quintana LAMP

The utility of the LAMP assay was tested by examining the blood samples of 100 rhesus macaque monkeys and 20 rhesus-feeders collected from the rhesus breeding base, Yibing City, Sichuan Province, China. qPCR and LAMP results were compared using the χ^2^ test and Fisher’s exact test.

## 5. Conclusions

In this study, a novel loop-mediated isothermal amplification (LAMP), which amplifies target DNA at a fixed temperature with high sensitivity, specificity and rapidity, was employed to detect *B. quintana*. According to the study, LAMP methodology is simpler than PCR-based methods and requires less equipment. It is a useful for diagnosis of trench fever in humans and primates, especially in low-resource settings, because of its rapid, sensitive detection that does not require sophisticated equipment.

## Figures and Tables

**Table 1:** The limit of detection (LOD) and time for LAMP and qPCR detection of *B. quintana groEL*.

Assay	Regions Recognized	LOD (DNA/Reaction)	Fastest Time (min)
LAMP	8	125 fg	18
qPCR	3	500 fg	35

**Table 2:** Detection of *B. quintana* in test samples of rhesus blood and rhesus-feeder blood by LAMP and qPCR.

Positive Rate (%) (No. of Positives/Total No. of Samples)		
Samples	LAMP	qPCR
100 rhesus	22.0 (22/100)	8.0 (8/100)
20 rhesus feeders	20.0 (4/20)	5.0 (1/20)

**Table 3:** Bacterial strains used in this study.

Bacterium	Source of Strain	No. of Strains
*B. quintana*	str. Toulouse	1
*B. quintana*	isolated strains (ICDC13001-13009)	9
*B. henselae*	isolated strains (ICDC14112)	1
*B. elizabethae*	isolated strains (ICDC14116)	1
*B. alsatica*	isolated strains (ICDC14117)	1
*B. koehlerae*	isolated strains (ICDC15002)	1
*B. vinsonii* subsp. *berkhoffii*	isolated strains (ICDC13010)	1
*B. vinsonii* subsp. *vinsonii*	isolated strains (ICDC10001)	1
*B. vinsonii* subsp. *arupensis*	isolated strains (ICDC10002)	1
*B. tribocorum*	isolated strains (ICDC11013)	1
*B. grahamii*	isolated strains (ICDC10161)	1
*B. clarridgeiae*	isolated strains (ICDC10181)	1
*B. bacilliformis*	isolated strains (ICDC13144)	1
*B. doshiae*	isolated strains (ICDC13167)	1
*B. mayotimonensis*	isolated strains (ICDC10009)	1
*Yersinia enterocolitica*	ATCC 23715	1
*Pseudomonas aeruginosa*	ATCC 15442	1
*Enterococcus faecalis*	ATCC 35667	1
*Aeromonas hydrophila*	ATCC 7966	1
*Enterobacter sakazakii*	ATCC 51329	1
*Campylobacter jejuni*	ATCC 33291	1
*Bacillus cereus*	Isolated strains (ICDC10118)	1
*Salmonella typhimurium*	Isolated strains (ICDC12113)	1
*Vibrio cholera*	Isolated strains (ICDC09111)	1
*Escherichia coli*	Isolated strains (ICDC08117)	1
*Listeria monocytogenes*	Isolated strains (ICDC12211)	1
*Shigella sonnei*	ATCC9372	1
*Staphylococcus aureus*	ATCC25923	1

ATCC, American Type Culture Collection; ICDC, National Institute for Communicable Disease Control and Prevention, China CDC.

**Table 4:** LAMP and qPCR primers used in this study.

Assay	Primer	Sequence (5′–3′)	Length (nt)
*groEL*_LAMP	F3	TGCTTTAGTCATTGGTTGTG	20
	B3	TTAATGAACTTTGGTGAGAGG	21
	FIP	ACAAACGCTGATATGCTTCTTTTTT-ATCAAGTCCTTGATTTAGAAGGT	48
	BIP	CATTCTCTTCATAGTGCAGTGGCT-ACGGACTGACATATGTGC	42
	LF	CTTGTGACTTTGTGAAAAATCTG	23
	LB	TCGGAATGGCCGAAAAATTTGG	22
*groEL*_qPCR	F	TGCCAAGTATGCGAGTTCCTG	21
	Probe	Cy5-TCTGCGCCCGGTTCTGAAATGCCT-BHQ2	24
	R	TCAGAGTTAGGTGCAAGTTCTATGG	25

## References

[1] Regier Y., O Rourke F., Kempf V.A. (2016). *Bartonella* spp.—A chance to establish one health concepts in veterinary and human medicine. Parasites Vectors.

[2] Minnick M.F., Anderson B.E., Lima A., Battisti J.M., Lawyer P.G., Birtles R.J. (2014). Oroya fever and verruga peruana: Bartonelloses unique to south america. PLoS Negl. Trop. Dis..

[3] Guy L., Nystedt B., Toft C., Zaremba-Niedzwiedzka K., Berglund E.C., Granberg F., Naslund K., Eriksson A.S., Andersson S.G. (2013). A gene transfer agent and a dynamic repertoire of secretion systems hold the keys to the explosive radiation of the emerging pathogen *Bartonella*. PLoS Genet..

[4] Foucault C., Brouqui P., Raoult D. (2006). *Bartonella* quintana characteristics and clinical management. Emerg. Infect. Dis..

[5] Breitschwerdt E.B., Maggi R.G., Nicholson W.L., Cherry N.A., Woods C.W. (2008). *Bartonella* sp. Bacteremia in patients with neurological and neurocognitive dysfunction. J. Clin. Microbiol..

[6] Kooli I., Loussaief C., Ben Brahim H., Aouem A., Toumi A., Chakroun M. (2014). *Bartonella quintana* meningoencephalitis in an immunocompetent: Rare case. Pathol. Biol. (Paris).

[7] Abromaitis S., Nelson C.S., Previte D., Yoon K.S., Clark J.M., DeRisi J.L., Koehler J.E. (2013). *Bartonella quintana* deploys host and vector temperature-specific transcriptomes. PLoS ONE.

[8] Pitassi L.H., de Paiva Diniz P.P., Scorpio D.G., Drummond M.R., Lania B.G., Barjas-Castro M.L., Gilioli R., Colombo S., Sowy S., Breitschwerdt E.B. (2015). *Bartonella* spp. Bacteremia in blood donors from campinas, brazil. PLoS Negl. Trop. Dis..

[9] Abromaitis S., Koehler J.E. (2013). The *Bartonella quintana* extracytoplasmic function sigma factor rpoe has a role in bacterial adaptation to the arthropod vector environment. J. Bacteriol..

[10] Bouhsira E., Ferrandez Y., Liu M., Franc M., Boulouis H.J., Biville F. (2013). Ctenocephalides felis an in vitro potential vector for five *Bartonella*species. Comp. Immunol. Microbiol. Infect. Dis..

[11] Chomel B.B., Kasten R.W., Henn J.B., Molia S. (2006). *Bartonella* infection in domestic cats and wild felids. Ann. N. Y. Acad. Sci..

[12] Leulmi H., Bitam I., Berenger J.M., Lepidi H., Rolain J.M., Almeras L., Raoult D., Parola P. (2015). Competence of cimex lectularius bed bugs for the transmission of *Bartonella quintana*, the agent of trench fever. PLoS Negl. Trop. Dis..

[13] Kernif T., Leulmi H., Socolovschi C., Berenger J.M., Lepidi H., Bitam I., Rolain J.M., Raoult D., Parola P. (2014). Acquisition and excretion of *Bartonella quintana* by the cat flea, ctenocephalides felis felis. Mol. Ecol..

[14] Angelakis E., Socolovschi C., Raoult D. (2013). *Bartonella quintana* in cimex hemipterus, rwanda. Am. J. Trop. Med. Hyg..

[15] Doern G.V. (2000). Detection of selected fastidious bacteria. Clin. Infect. Dis..

[16] Chamberlin J., Laughlin L., Gordon S., Romero S., Solorzano N., Regnery R.L. (2000). Serodiagnosis of *Bartonella bacilliformis* infection by indirect fluorescence antibody assay: Test development and application to a population in an area of bartonellosis endemicity. J. Clin. Microbiol..

[17] Sanchez Clemente N., Ugarte-Gil C.A., Solorzano N., Maguina C., Pachas P., Blazes D., Bailey R., Mabey D., Moore D. (2012). *Bartonella bacilliformis*: A systematic review of the literature to guide the research agenda for elimination. PLoS Negl. Trop. Dis..

[18] Wang Y., Wang Y., Xu H., Dai H., Meng S., Ye C. (2014). Rapid and sensitive detection of listeria ivanovii by loop-mediated isothermal amplification of the smcl gene. PLoS ONE.

[19] Tomita N., Mori Y., Kanda H., Notomi T. (2008). Loop-mediated isothermal amplification (LAMP) of gene sequences and simple visual detection of products. Nat. Protoc..

[20] Chen Z., Liao Y., Ke X., Zhou J., Chen Y., Gao L., Chen Q., Yu S. (2011). Comparison of reverse transcription loop-mediated isothermal amplification, conventional PCR and real-time PCR assays for japanese encephalitis virus. Mol. Biol. Rep..

[21] Angkasekwinai N., Atkins E.H., Johnson R.N., Grieco J.P., Ching W.M., Chao C.C. (2014). Rapid and sensitive detection of *Bartonella bacilliformis* in experimentally infected sand flies by loop-mediated isothermal amplification (LAMP) of the *pap31* gene. PLoS Negl. Trop. Dis..

[22] Notomi T., Okayama H., Masubuchi H., Yonekawa T., Watanabe K., Amino N., Hase T. (2000). Loop-mediated isothermal amplification of DNA. Nucleic Acids Res..

[23] Liberto M.C., Lamberti A.G., Marascio N., Matera G., Quirino A., Barreca G.S., Baudi F., Foca A. (2011). Molecular identification of *Bartonella quintana* infection using species-specific real-time PCR targeting transcriptional regulatory protein (*BQTR*) gene. Mol. Cell. Probes.

[24] Sato S., Kabeya H., Yoshino A., Sekine W., Suzuki K., Tamate H.B., Yamazaki S., Chomel B.B., Maruyama S. (2015). Japanese macaques (*macaca fuscata*) as natural reservoir of *Bartonella quintana*. Emerg. Infect. Dis..

[25] Zeaiter Z., Liang Z., Raoult D. (2002). Genetic classification and differentiation of *Bartonella* species based on comparison of partial *ftsz* gene sequences. J. Clin. Microbiol..

[26] Korhonen E.M., Perez Vera C., Pulliainen A.T., Sironen T., Aaltonen K., Kortet R., Harkonen L., Harkonen S., Paakkonen T., Nieminen P. (2015). Molecular detection of *Bartonella spp*. In deer ked pupae, adult keds and moose blood in finland. Epidemiol. Infect..

[27] Lee D., Kim E.J., Kilgore P.E., Kim S.A., Takahashi H., Ohnishi M., Anh D.D., Dong B.Q., Kim J.S., Tomono J. (2015). Clinical evaluation of a loop-mediated isothermal amplification (LAMP) assay for rapid detection of neisseria meningitidis in cerebrospinal fluid. PLoS ONE.

[28] Pan L., Zhang L., Fan D., Zhang X., Liu H., Lu Q., Xu Q. (2013). Rapid, simple and sensitive detection of q fever by loop-mediated isothermal amplification of the *htpab* gene. PLoS Negl. Trop. Dis..

[29] Paris D.H., Blacksell S.D., Newton P.N., Day N.P. (2008). Simple, rapid and sensitive detection of orientia tsutsugamushi by loop-isothermal DNA amplification. Trans. R. Soc. Trop. Med. Hyg..

[30] Iseki H., Alhassan A., Ohta N., Thekisoe O.M., Yokoyama N., Inoue N., Nambota A., Yasuda J., Igarashi I. (2007). Development of a multiplex loop-mediated isothermal amplification (MLAMP) method for the simultaneous detection of bovine babesia parasites. J. Microbiol. Methods.

[31] Lau Y.L., Fong M.Y., Mahmud R., Chang P.Y., Palaeya V., Cheong F.W., Chin L.C., Anthony C.N., Al-Mekhlafi A.M., Chen Y. (2011). Specific, sensitive and rapid detection of human plasmodium knowlesi infection by loop-mediated isothermal amplification (LAMP) in blood samples. Malar. J..

[32] Minnick M.F., Smitherman L.S., Samuels D.S. (2003). Mitogenic effect of *Bartonella bacilliformis* on human vascular endothelial cells and involvement of groel. Infect. Immun..

